# Air Bag Deployment With an Aftermarket Steering Wheel Decoration Resulting in a Penetrating Intracranial Injury

**DOI:** 10.7759/cureus.91292

**Published:** 2025-08-30

**Authors:** Jose E Marino, Felipe Monteiro, Danny L John, Joacir G Cordeiro, Ronald Benveniste

**Affiliations:** 1 Neurological Surgery, University of Miami Miller School of Medicine, Jackson Memorial Hospital, Miami, USA

**Keywords:** air bag deployment, craniotomy, intracranial foreign object, penetrating intracranial injury, post-traumatic epilepsy, steering wheel object

## Abstract

We present a rare case of penetrating brain injury caused by an aftermarket steering wheel decoration during air bag deployment. A female driver was involved in a collision during which the driver’s air bag deployed, propelling a crescent-shaped, sequin-covered decorative object into her face. The object penetrated the right globe and orbital roof, lodging in the right frontal lobe of her brain. Imaging confirmed the presence of an intracranial foreign body without vascular injury. The patient underwent successful globe repair and craniotomy for foreign body removal. She remained neurologically intact postoperatively with preserved light perception in the injured eye. This case highlights a rare but serious risk associated with steering wheel decorative objects and highlights the need for greater public education and consideration of regulatory measures to prevent similar injuries in the future.

## Introduction

Air bags are a key component of vehicular safety systems and have saved more than 50,000 lives over a 30-year period. However, their deployment can occasionally result in severe and, rarely, fatal injuries [1]. Injuries include facial and spinal trauma due to the impact of the deployed air bag, particularly in small or unrestrained adults and in children. In some cases, metal fragments from defective chemical cannisters responsible for air bag deployment caused penetrating injuries to the eyes, face, and neck and led to a massive recall of air bags produced by a specific manufacturer [2,3]. More recently, the National Highway Traffic Safety Administration (NHTSA) reported penetrating injuries to the eye and face caused by aftermarket decorations glued to the steering wheel and advised the public not to use these products [4]. Modern cars have air bags stored under the steering wheel cover, and aftermarket decorations in that location can become high velocity projectiles upon air bag deployment. Previous reports of penetrating injuries related to steering wheel decorations described intraorbital injuries. To our knowledge, this appears to be a rare case of a steering wheel decoration penetrating the brain after air bag deployment. This case emphasizes and adds to concerns about the public health risk of these products and highlights the need for public awareness and possibly legislation to prevent future injuries.

## Case presentation

**Figure 1:**
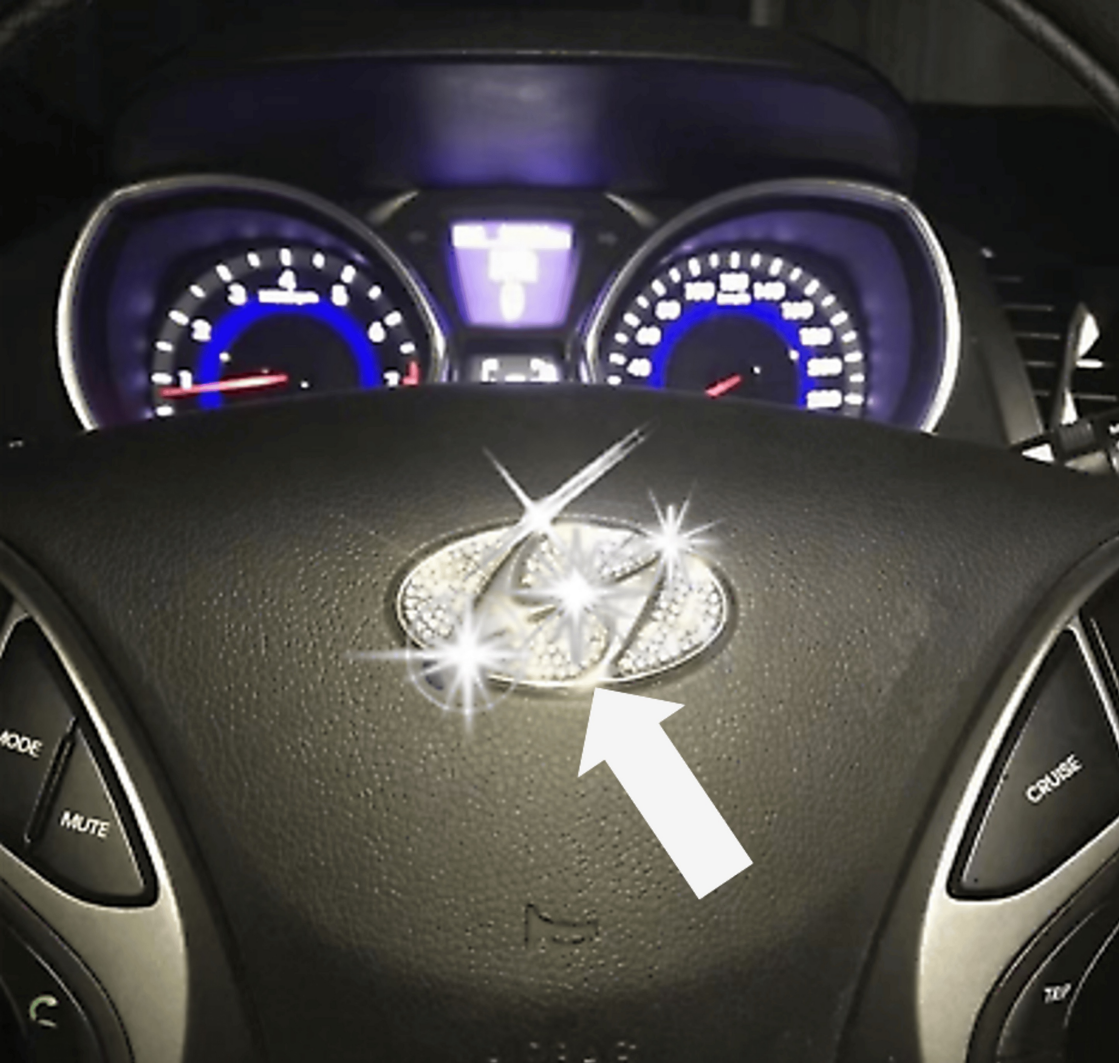
Steering wheel decoration Steering wheel decoration consisting of the vehicle manufacturer’s logo, bedazzled with rhinestones and adhered to the center of the steering wheel using glue. This aftermarket embellishment was positioned directly over the air bag cover. Upon air bag deployment, the decoration became a high-velocity projectile, resulting in craniofacial penetration

The patient is an adult woman in her late forties who was the driver of a car that struck a deer on the highway. The driver-side air bag, located in the steering wheel cover, deployed. On presentation, the patient was alert and conversant. She had a facial laceration with an embedded crescent-shaped foreign body covered with sequins. The patient reported that the object came from a decoration she had glued to her steering wheel (Figure 1).

**Figure 2:**
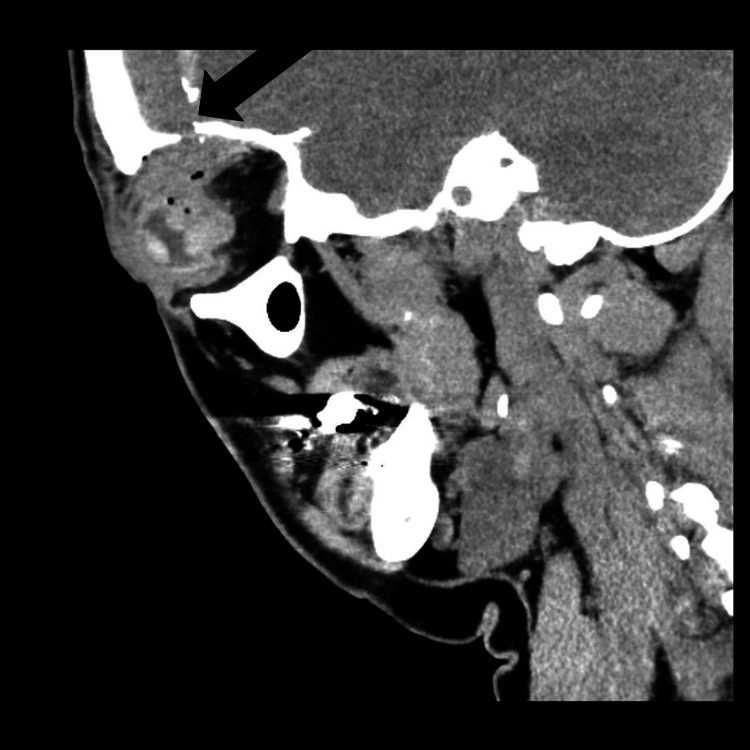
Orbital roof defect with bone fragments and hematoma on sagittal CT CT: computed tomography Sagittal CT images show a defect in the orbital roof, with displaced bone fragments and hematoma along the path of a foreign body (not visualized in this image), indicating traumatic penetration

**Figure 3:**
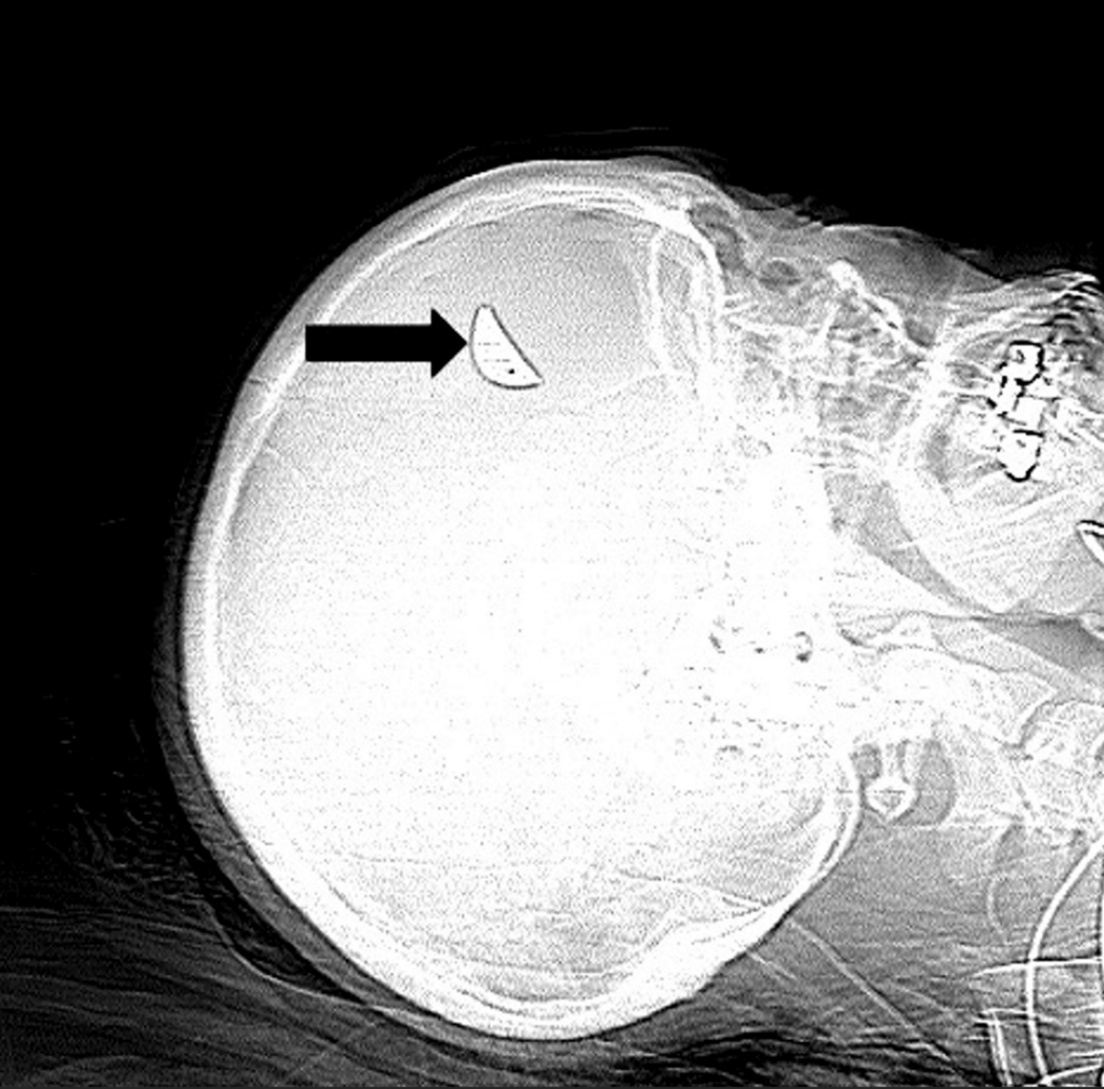
Scout image of the intracranial foreign body preoperatively Preoperative scout X-ray image demonstrating a dense, crescent-shaped intracranial foreign body measuring approximately 2 cm, located in the dorsolateral aspect of the right frontal lobe

The patient was also found to have a penetrating injury to the right globe, resulting in visual loss. She was transferred from a local hospital to our tertiary care center for further evaluation and management. Ophthalmology evaluation revealed a rupture of the right globe, with only light perception and an afferent pupillary defect. The patient remained alert and otherwise neurologically intact. CT of the brain showed a breach in the right orbital roof with small bone fragments in the basal right frontal lobe (Figure 2) and a dense, 2 cm, crescent-shaped foreign body in the dorsolateral right frontal lobe (Figure 3).

**Figure 4:**
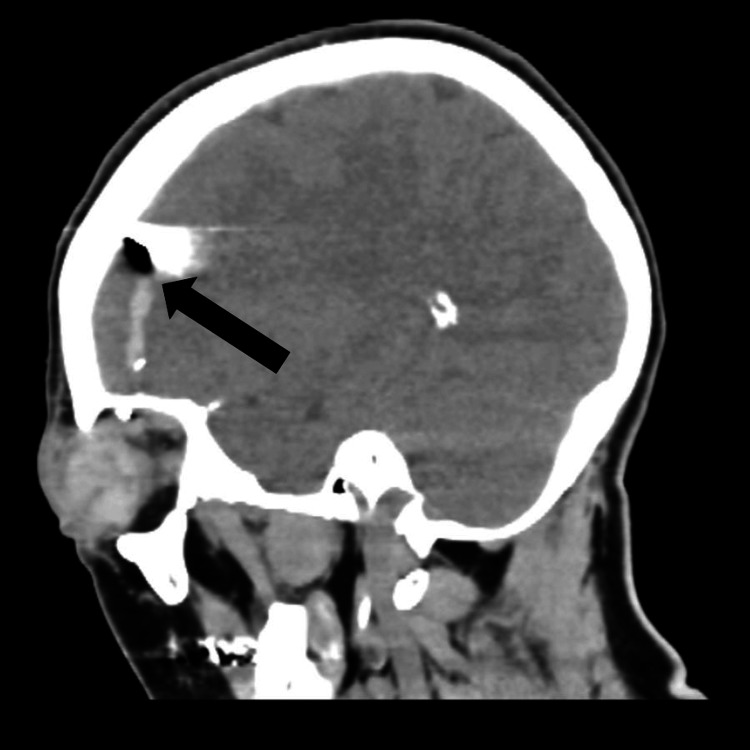
Intracranial foreign body and associated hematoma following air bag deployment: sagittal CT imaging CT: computed tomography Sagittal CT images reveal a small intracranial hematoma, located just caudal to a more rostrally positioned foreign body. The injury occurred following air bag deployment, with the foreign object penetrating the cranial cavity and resulting in localized hemorrhage

**Figure 5:**
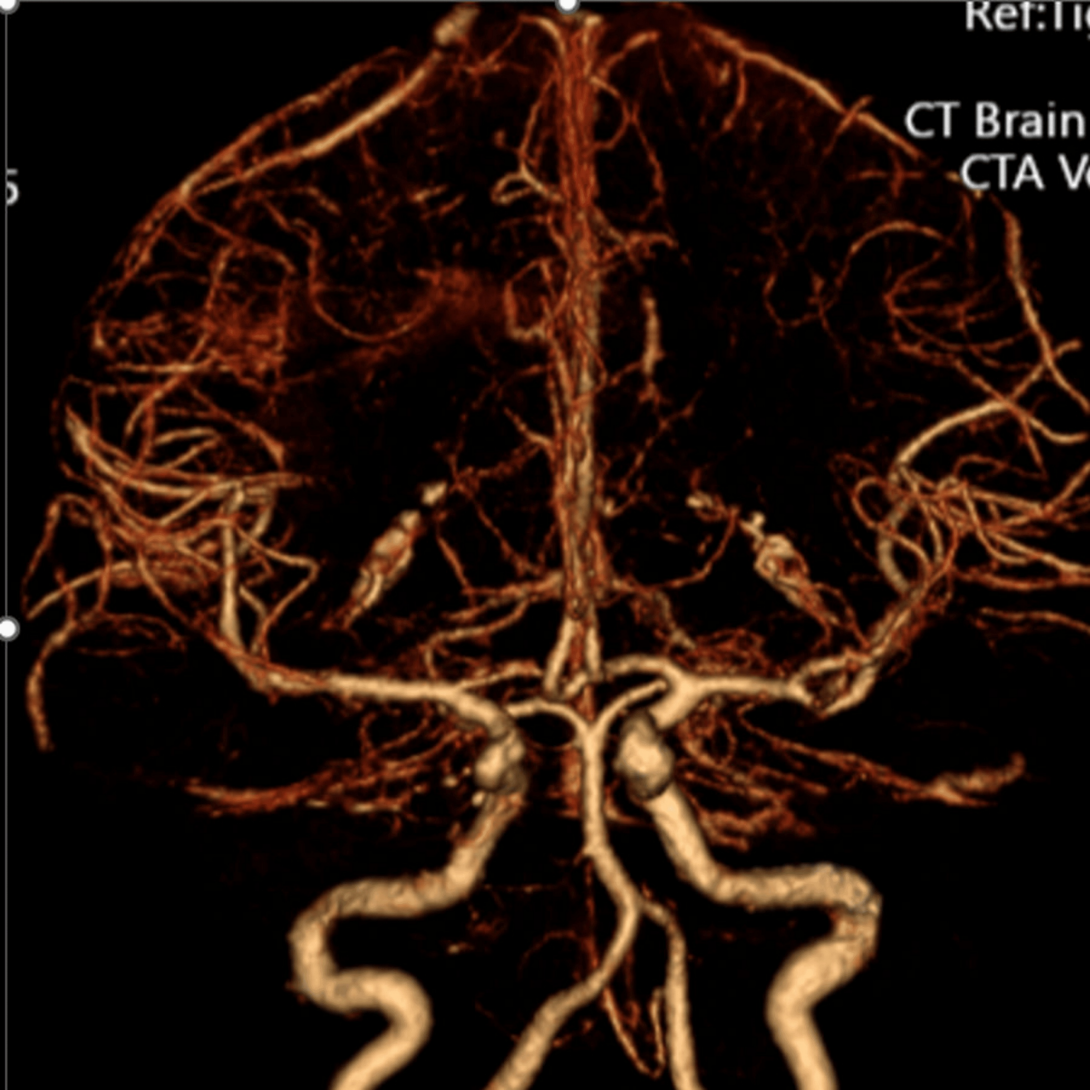
CTA head with contrast CTA: computed tomography angiography CTA of the head with contrast demonstrating normal intracranial vasculature. There is no evidence of large vessel occlusion, critical stenosis, aneurysm, or traumatic pseudoaneurysm

**Figure 6:**
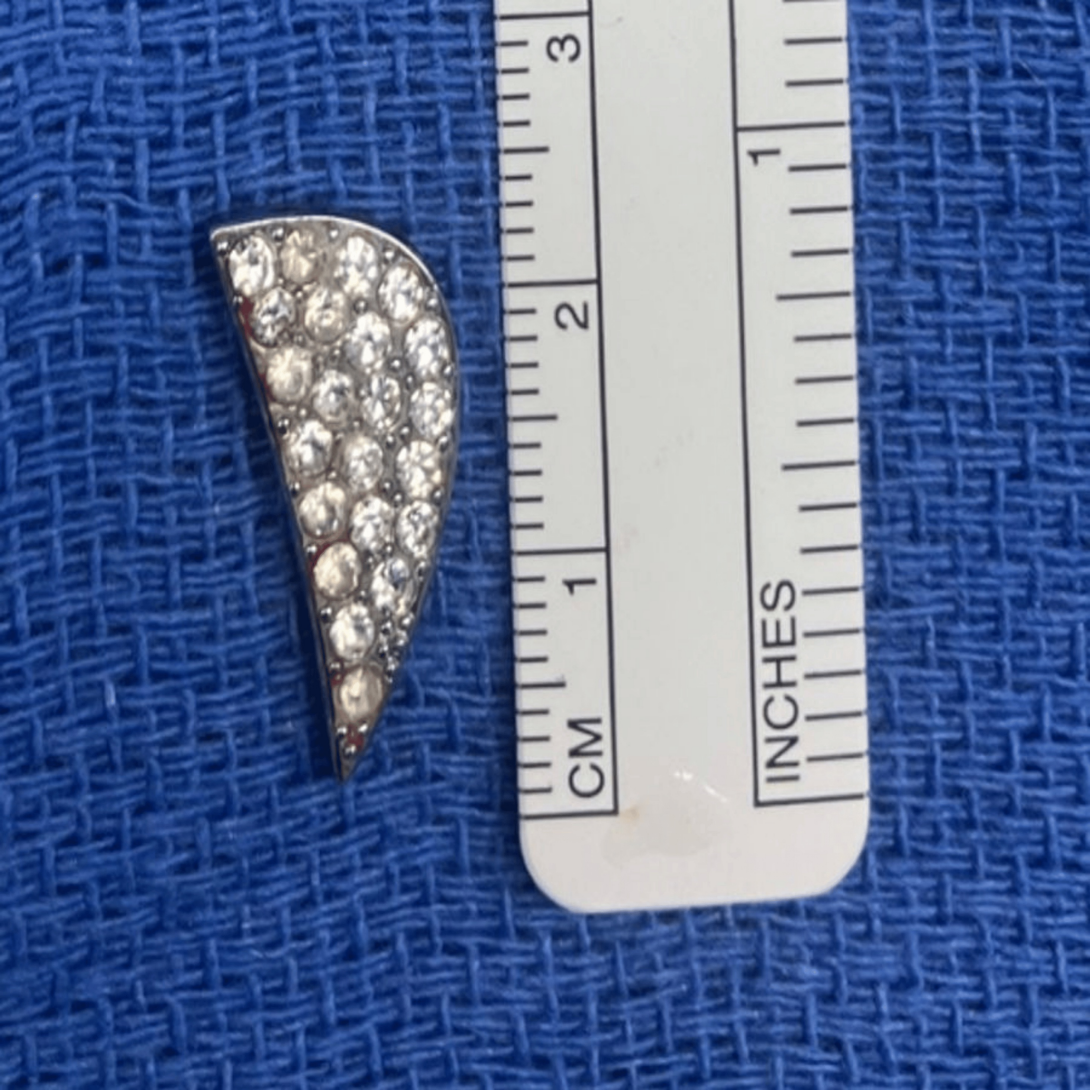
Foreign body intraoperatively Intraoperative photograph of the retrieved intracranial foreign body, measuring 2 cm in length. The object consists of a metallic fragment from a vehicle manufacturer’s logo, bedazzled with rhinestones. This decorative emblem had been glued to the center of the steering wheel and became a projectile during air bag deployment

Preoperative sagittal CT images demonstrated a small intracranial hematoma located just caudal to a more rostrally positioned foreign body (Figure 4). Repeat head CT showed no new or worsened hemorrhage, and CT angiography of the brain showed no arterial injury (Figure 5). When informed about the intracranial foreign body, the patient stated that she believed it came from a decoration on her steering wheel and that another fragment had been removed from a facial laceration at the initial hospital. The patient received prophylactic vancomycin 1 gram twice daily and ceftriaxone 2 grams every 12 hours for 10 days. On hospital day 2, she underwent repair of her ruptured globe and postoperatively retained light perception in the right eye. On hospital day 3, she underwent a right frontal craniotomy and removal of the intracranial foreign body to minimize the risk of infection. The foreign body was found just beneath a small linear tear in the dura, presumably caused by the foreign body itself, and was surrounded by a small amount of necrotic brain tissue. The foreign body was covered with sequins and matched the patient’s description of her steering wheel cover decoration (Figure 6).

**Figure 7:**
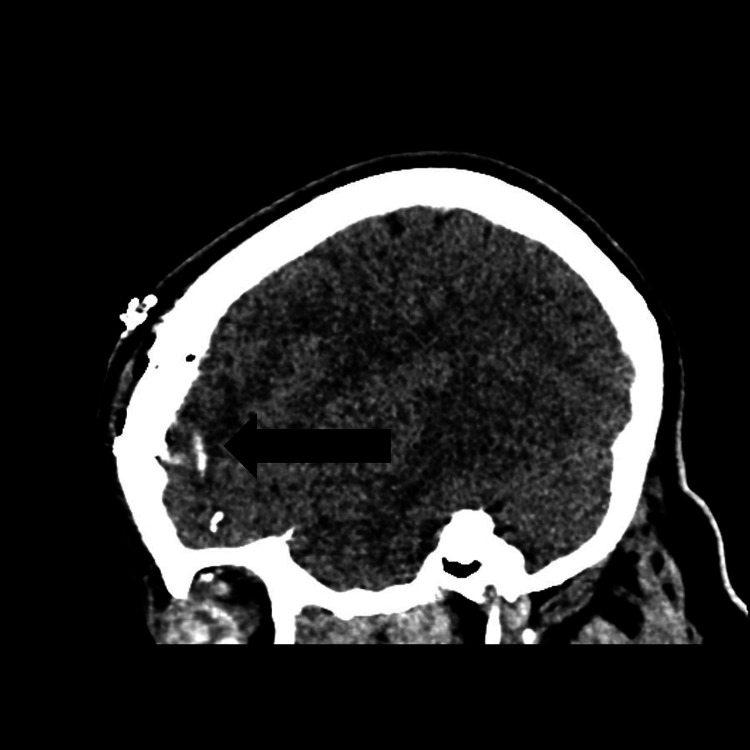
Postoperative sagittal CT showing residual hematoma after foreign body removal CT: computed tomography Postoperative sagittal CT image demonstrates residual hematoma along the track of the removed foreign body, confirming successful extraction and highlighting the area of localized hemorrhage

Postoperatively, the patient remained neurologically intact, with light perception in the right eye and no seizures. Postoperative CT showed no new hemorrhage or other complications (Figure 7). The patient completed a course of prophylactic broad-spectrum antibiotics and was discharged to inpatient rehabilitation on prophylactic anticonvulsants (Keppra 1 gram twice daily) due to the presence of small, retained bone fragments in the basal frontal lobe.

## Discussion

Driver and passenger side air bags were mandated by the Intermodal Surface Transportation Efficiency Act of 1991 for all passenger cars and light trucks in the United States manufactured after September 1, 1998. Although air bags reduce overall motor vehicle accident-related morbidity and mortality, injuries related to their deployment do occur. A subset of these injuries relates to ejection of high-velocity metal fragments from the metal cannisters storing the chemicals needed for rapid air bag inflation, causing penetrating injuries to the face, neck, eyes, and upper limbs [1,5,6].

In one well-publicized case, several million air bags produced by a single manufacturer were recalled due to a defect in the inflator mechanism caused by degradation of chemicals in the inflator after exposure to high temperature and humidity, resulting in excessive pressure and the ejection of metal fragments from the cannisters, in some cases causing fatal injuries [3].

Although most reported penetrating injuries were related to defective air bag inflators, a recent warning from the NHTSA warned consumers about the risk of penetrating injuries caused by aftermarket steering wheel decorations that are glued to the steering wheel cover and that could become detach and become projectiles when the underlying air bag deploys. At least one driver suffered a serious injury that resulted in the loss of sight in one eye, when an aftermarket emblem adorned with rhinestones dislodged from the steering wheel in a crash and hit the driver in the face [4].

We report a case of penetrating injury to the globe and frontal lobe of the brain, caused by dislodgment of an aftermarket steering wheel cover decoration during deployment of an air bag. A large fragment of the decoration passed through the orbit and thin bone of the orbital roof, traversing the frontal lobe of the brain. Given the relative resistance of the globe and amount of velocity that the fragment needed in order to penetrate the orbital roof and brain, we hypothesize that the fragment passed along the surface of the sclera before penetrating the orbital roof [7,8]. Importantly, the patient’s only injuries were the orbital and intracranial trauma caused by this foreign body; without this event, she would likely have remained uninjured.

Fortunately, our patient avoided the most significant complications related to penetrating brain trauma, including hemorrhage causing mass effect or neurological deficits, neurovascular injury, and meningitis or abscess formation [9,10].

Management of our patient’s combined orbital and intracranial trauma was straightforward and involved repair of her ruptured globe by our ophthalmologists, removal of her intracranial foreign body by our neurosurgeons, and medications to minimize the risk of seizure and infection [11].

## Conclusions

This case highlights the risks of aftermarket steering wheel decorations, including demonstration of a previously unreported risk of air bag deployment. It supports the National Transportation Safety Board (NTSB) recommendations to avoid the aftermarket steering wheel embellishments, particularly those that may become projectiles during air bag deployment. These findings presented here showed a preventable and serious mechanism of injury reinforcing the need for stronger public safety measures. Moreover, this case raises the question of whether further legislative actions such as regulation by the Consumer Product Safety Commission may be necessary to restrict the sale and distribution of these products.
